# The Book World of Medicine and Science

**Published:** 1897-07-24

**Authors:** 


### THE BOOK WORLD OF MEDICINE AND SCIENCE.

B. Bradshaw's Dictionary of Mineral Waters, Climatic Health Resorts, Sea Baths, and Hydropathic Establishments, 1897. (London: Kegan Paul, Trench, and Co. Price 2s. 6d.)

This is a little book which is likely to be of much use to those who want the information contained in it, while to those who are not interested in baths and waters and care little about their livers it will be but dull reading. The number of watering-places, however, is so great, and the multitude of those who wish to derive benefit from them, but do not know where to go, is so enormous that we think it very probable that this book will have a considerable success. At the same time it must be admitted that the information given about the majority of these places is but small, and unfortunately it is just in regard to the little known places that the information given is most scanty, while those which are dealt with in greatest detail are those about which most people at least know something. We have looked carefully through a considerable number of the articles, and, so far as regards places which are known to us, they seem up to date and free from error. The list of diseases and places where they are most effectually treated is a thing with which some medical men will perhaps find fault. It might be better to substitute the word "commonly" for "effectually," but after all the effectiveness of baths and waters in any particular disease depends so much upon the appliances being properly arranged for the treatment of the disease in question that we do not feel inclined to quarrel even with this chapter, which seems the only one to which anyone will be likely to take exception. As a reference book on the physician's table this volume will certainly be of service, although that probably is not the purpose for which it was written.

